# Bystander Selection for Antimicrobial Resistance: Implications for Patient Health

**DOI:** 10.1016/j.tim.2019.06.004

**Published:** 2019-07-06

**Authors:** Valerie J. Morley, Robert J. Woods, Andrew F. Read

**Affiliations:** 1 Center for Infectious Disease Dynamics, Departments of Biology and Entomology, The Pennsylvania State University, University Park, PA, USA; 2 Division of Infectious Diseases, Department of Internal Medicine, University of Michigan, Ann Arbor, MI, USA; 3 Huck Institutes for the Life Sciences, The Pennsylvania State University, University Park, PA, USA

## Abstract

Antimicrobial therapy promotes resistance emergence in target infections and in off-target microbiota. Off-target resistance emergence threatens patient health when off-target populations are a source of future infections, as they are for many important drug-resistant pathogens. However, the health risks of antimicrobial exposure in off-target populations remain largely unquantified, making rational antibiotic stewardship challenging. Here, we discuss the contribution of bystander antimicrobial exposure to the resistance crisis, the implications for antimicrobial stewardship, and some novel opportunities to limit resistance evolution while treating target pathogens.

## Off-Target Antimicrobial Exposure

Antimicrobial-resistant infections cause more morbidity, more mortality, longer hospital stays, and higher healthcare costs than do antimicrobial-susceptible infections [1]. **Antimicrobial stewardship** (see Glossary) practices aim to slow resistance emergence within patients and the spread of resistant microbes between patients. Some popular stewardship practices are intended to reduce the antimicrobial exposure experienced by **bystander** organisms in the patient’s broader microbiome. Bystanders are organisms that are not the **targets** of treatment. Stewardship practices intended to reduce bystander exposure include the avoidance of unnecessary antimicrobial use (e.g., for viral infections) and preferential use of narrow-spectrum antimicrobials [2].

There are two reasons to protect **off-target** microbes from antimicrobial exposure. First, off-target antimicrobial exposure can disrupt the normal **microbiota**, which can have health consequences, including the loss of **colonization resistance** [3]. Second, off-target exposure can promote antimicrobial resistance evolution within the patient’s **microbiome** [4–18]. Here, we focus on that evolution and its health consequences. Enrichment for antimicrobial resistance in a patient’s microbiome could increase the risk that the patient will subsequently have a resistant infection. Off-target enrichment for resistance could also increase the transmission of resistant organisms between individuals [19]. Here, we focus on how off-target exposure influences evolution and health risks within a patient.

## Antimicrobial Therapy Enriches for Resistance in Off-Target Microbial Populations

**Figure 1.**
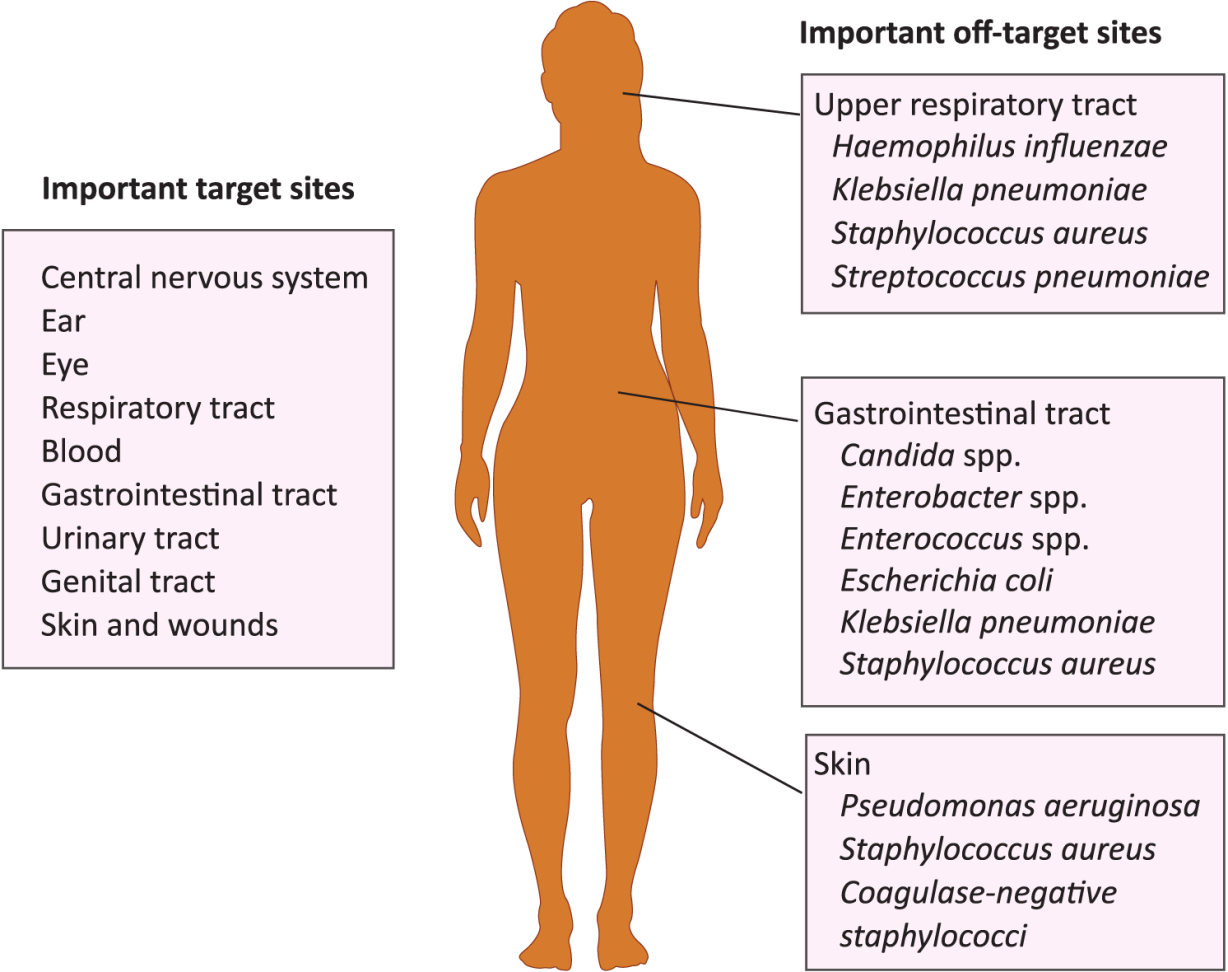
Important Sites for Target and Off-Target Antimicrobial Exposure. Colonizing species of greatest concern are listed for off-target sites.

The human body hosts a diverse microbiota that includes trillions of bacteria [3,20]. Microbial community composition varies across anatomical sites [3], with the largest bacterial populations found on the skin, in the oral cavity, and in the gastrointestinal (GI) tract [20]. Microbiome composition varies extensively among individuals and is influenced by environmental factors such as diet and antimicrobial exposure [3]. Most bacteria in the microbiome are **commensal**, meaning that they do not harm their host, and some provide benefits to host metabolism or other host functions [3]. Pathogenic organisms infect human hosts at many different anatomical sites (Figure 1). To successfully treat an infection, an antimicrobial must reach microbes at the target infection site. The antimicrobial might also affect the microbiome at off-target sites (Figure 1).

Physiological drug concentrations depend on the drug, the dose administered, patient characteristics (e.g., body mass or renal function), administration route (e.g., oral or intravenous), plasma protein binding, and rates of tissue penetration and clearance [21]. The result is different concentrations of antimicrobial at different anatomical sites during therapy, which creates a diversity of antimicrobial environments within the body. When antimicrobials reach sites hosting microbiota, they affect bacteria in several ways. Subinhibitory concentrations can alter gene expression in bacteria, sometimes inducing expression of facultative resistance genes or increasing rates of **conjugation** [22]. Most obviously, and the focus of this review, antimicrobials exert selection for resistance, and if a microbial population includes resistant variants, these variants will generally increase in frequency. These dynamics can be complicated by interspecies interactions, including cross-feeding [23,24] and loss of colonization resistance, which allow resistant organisms to colonize the body. In practice, the relative contributions of expansion of endogenous resistant genotypes and colonization with exogenous resistant organisms often are not determined.

Overwhelmingly, data show that antimicrobial exposure causes increased antimicrobial resistance in the microbiome [4–18]. Most studies have focused on the gut microbiota [5–13,16,18], but others have tracked resistance in the pharyngeal [4,5,14], nasal [15,17], skin [25], and oral [26] microbiota. Links between antimicrobial exposure and increased resistance in the microbiome have been shown for many classes of drugs, including β-lactams [4,7,8,13,26], macrolides [14,16,17], fluoroquinolones [5,10,15,25], tetracyclines [13], lincosamides [12], sulfonamides [11,18], and quinolones [9]. Bacteria demonstrating increased resistance come from many families, including Pasteurellaceae [4], Enterobacteriaceae [5,7–10,13,18], Streptococcaceae [5,14,15], Bacteroidaceae [12], Enterococcaceae [16], and Staphylococcaceae [17].

Resistance in the microbiome typically peaks near the end of antimicrobial treatment, usually returning to baseline in the subsequent weeks or months [6,14,27]. A recent systematic review evaluated the persistence of resistance in patients prescribed antimicrobials in primary care. Immediately following antimicrobial therapy, they found patient microbiomes generally harbored elevated resistance. For some antibiotic classes and focal bacteria, resistance levels decayed to baseline 1–3 months after treatment, while for others, no decay was seen within the sampling period (usually no more than 3 months) [27]. For example, patients treated with penicillin-class antibiotics were four times as likely to carry resistant *Streptococcus pneumoniae* in the respiratory tract compared with untreated patients immediately following treatment. The number of patients carrying resistant bacteria decreased after 1 month, but still had not returned to baseline. Three months after macrolide exposure, treated patients were still at least twice as likely to carry resistant *S. pneumoniae* compared with untreated patients. Some studies have reported elevated resistance in the microbiome persisting for months or years following treatment [12,14,16,17,28,29].

Commensal bacteria suppress intestinal opportunistic pathogens by modulating the host immune system and by competitive exclusion [30]. This phenomenon is called colonization resistance. By disrupting the intestinal microbiota, antimicrobials promote the invasion and proliferation of resistant bacteria not typically numerous in the gut [30]. Clinically important pathogens such as *Clostridium difficile* and vancomycin-resistant *Enterococcus* successfully colonize these disrupted environments [30]. Off-target antimicrobial exposure paves the way for these opportunists to proliferate, and it exerts selection for resistance in these expanded populations. For example, in England, rates of infection with fluoroquinolone-resistant *C. difficile* are closely tied to total regional rates of fluoroquinolone prescribing [31].

The enrichment of resistance genes in off-target populations could put the patient at risk if (i) resistant organisms from off-target populations subsequently cause symptomatic infections, or if (ii) resistance genes in off-target populations are **horizontally transferred** to pathogens. We take each of these in turn.

## Colonizing Opportunistic Pathogens: Bystanders Posing a Risk to Patient Health

**Colonizing opportunistic pathogens (COPs)** are organisms that colonize the body asymptomatically, but cause disease in immunocompromised patients or when introduced to other anatomical sites [32]. For example, *S. pneumoniae* asymptomatically colonizes the upper respiratory tract in up to 65% of children and up to 10% of adults, but these bacteria can cause otitis media, sepsis, pneumonia, or meningitis when introduced to other anatomical sites [33]. Similarly, *Enterococcus faecium* and *Klebsiella pneumoniae* colonize the GI tract asymptomatically, but cause infections when introduced to the bloodstream or urinary tract [34,35]. Many important drug-resistant pathogens are COPs (Table 1).

Colonization with COPs is a risk factor for infection [35–39] because patients can become infected by the COPs they carry [33,35,36,38–42]. At the University of Michigan hospital, patients whose GI tracts were colonized with *K. pneumoniae* were on average four times more likely to get *Klebsiella* infections than those who were colonization-negative, and in most cases (81%), the infecting strain matched the patient’s colonizing strain [35]. A study of intensive care unit (ICU) patients in Melbourne reported similar results [36]. Similarly, numerous clinical studies found that nasal colonization with *Staphylococcus aureus* increased the risk of nosocomial *S. aureus* bacteremia, with reported relative risks ranging from 1.2 to 21.7 [37–39]. The majority of *S. aureus* infections in colonized patients matched the colonizing strain [38,39,41]. Colonizing populations are also sources of infections for enterococci [43], extraintestinal pathogenic *Escherichia coli* (ExPEC) [40], *S. pneumoniae* [33], *Haemophilus influenzae* [44], and *Candida albicans* [42]. Colonizing and infecting strains within a single patient often (but not always) match.

For many COPs, most of their antimicrobial exposure occurs when they are not the targets of treatment. One study estimated that, in the USA, over 90% of the total antimicrobial exposure experienced by *K. pneumoniae* occurred when *K. pneumoniae* was not the target pathogen [2]. This included exposure to antimicrobials relevant to treating *K. pneumoniae* infections, such as penicillins and tetracyclines. For *H. influenzae*, *E. coli*, *S. pneumoniae*, *S. aureus*, and *Pseudomonas aeruginosa*, over 80% of total exposure to antibiotics was estimated to occur when the bacteria were bystanders [2]. This off-target antimicrobial exposure demonstrably selects for resistance in colonizing populations. Off-target antimicrobial exposure has been shown to select for resistance in *K. pneumoniae* populations colonizing the guts of infants [45] and in staphylococci on the skin [46,47] and in the nasal cavity [15,17].

Putting these lines of evidence together, it seems highly likely that enriching for resistance in commensal COP populations increases a patient’s risk of a subsequent resistant infection originating from their own flora: patients carrying a COP are more likely to become infected with that COP; COPs experience most exposure to antibiotics when they are bystanders, and bystander exposure can make the COP resistant. However, we know of no direct evidence that a COP became resistant as a bystander, and then went on to become a resistant infection. Such data in a single patient would require longitudinal sampling of commensal bacteria in an individual patient beginning prior to a course of antibiotic therapy and extending through subsequent infections. We note that, while this review focuses on bystander selection in patients, many opportunistic pathogens also have reservoirs in environmental or animal populations, and these reservoirs are likely also important sites for resistance evolution [48].

## Horizontal Transfer of Resistance from Off-Targets to Pathogens

**Figure 2.**
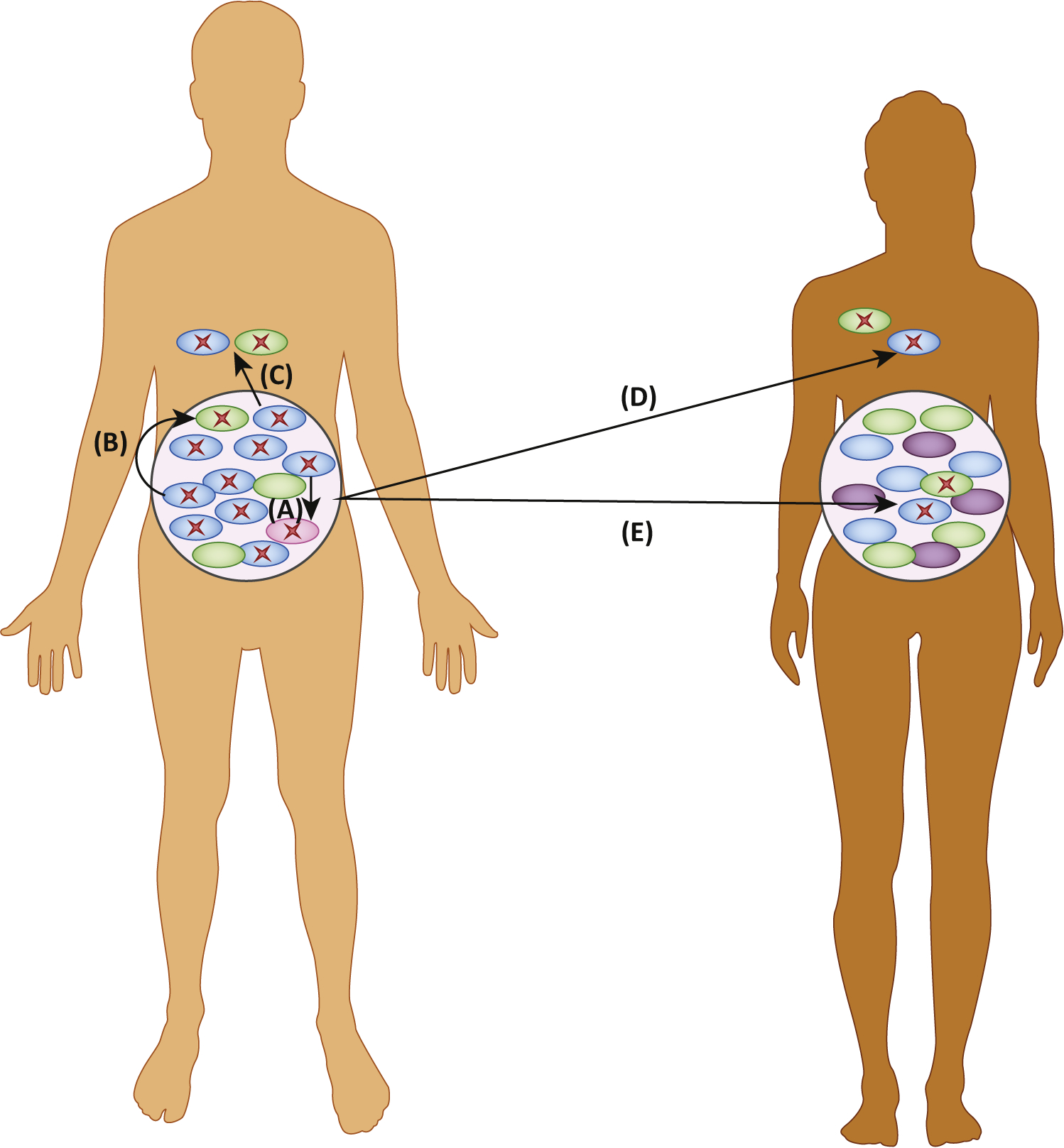
The Risks of Enrichment for Antimicrobial-Resistance Genes in Off-Target Microbial Populations. (A) If the patient acquires an obligate pathogen (pink), the pathogen can acquire resistance genes (red X) from other bacteria in the microbiome (green and blue). (B) Resistance genes can be horizontally transferred between strains and between species. This can produce resistant colonizing opportunistic pathogens (COPs). (C) Resistant organisms from the microbiome can cause opportunistic infections at other sites in the patient’s body. (D) Resistant organisms can transmit and opportunistically infect another patient. (E) Resistant organisms can colonize another patient asymptomatically.

Resistance determinants in the microbiome can transfer horizontally between bacteria. This poses a risk to patient health when it produces resistant bacteria that go on to cause symptomatic infections (Figure 2). Bacteria have three main mechanisms of **horizontal gene transfer (HGT)**: conjugation, **transformation**, and **transduction** [53]. All of these mechanisms can transfer antibiotic resistance determinants among bacteria. The likelihood of transfer between bacteria is correlated with phylogenetic relatedness, with more closely related bacteria being more likely to transfer genes [54]. Barriers to HGT include recipient restriction enzyme activity, bacteriophage host range, limits on development of natural competence, limits on the host range of plasmid transfer mechanisms, CRISPR interference, and requirements for sequence similarity to integrate foreign DNA into a replicating genetic element [53].

The microbiome is a hotspot for recombination of resistance genes into new genetic backgrounds. Human-associated bacteria horizontally transfer genes 25-fold more frequently than bacteria in other aquatic or terrestrial environments [55]. Studies in animal models [56–59] and humans [60,61] have demonstrated transfer of resistance determinants among bacteria in the gut [56–60] and the respiratory tract [61]. Antimicrobial exposure further increases rates of horizontal transfer. Subinhibitory antimicrobial exposure induces bacteriophages to transition from lysogeny to lysis, which increases gene transfer by transduction [62]. Antimicrobials can also induce the **SOS response**, which promotes mutagenesis and can increase conjugation [63–66].

Historically, HGT has played a major role in the spread of clinically important resistance genes. For example, at least 23 *S. aureus* lineages have independently acquired SCC*mec*, a mobile element conferring resistance to practically all β-lactam antibiotics, either from another methicillin-resistant *S. aureus* (MRSA) lineage or from coagulase-negative *Staphylococcus* (CoNS) species [67]. CoNS and *S. aureus* often cocolonize hosts at the same anatomical sites, making the microbiome an important site for potential SCC*mec* transfer. The transfer of plasmids in the microbiome also contributes to the spread of vancomycin resistance in enterococci and staphylococci [68,69]. Transferable plasmids carry other important antimicrobial resistance determinants, including β-lactamases, *mcr-1*, *optrA*, and *qnrA* [70,71].

Transfer of resistance determinants produces new resistant strains, and even shuffles resistance determinants between species. When off-target antimicrobial exposure enriches for organisms carrying resistance genes, it may increase the potential for horizontal transfer of resistance genes within the patient. Barriers to HGT mean that interspecies transmissions of resistance genes are relatively rare events, but a single transfer has the potential to be disastrous if the clone amplifies and spreads.

## Prior Off-Target Antimicrobial Exposure as a Risk Factor for Resistant Infections

**Figure 3.**
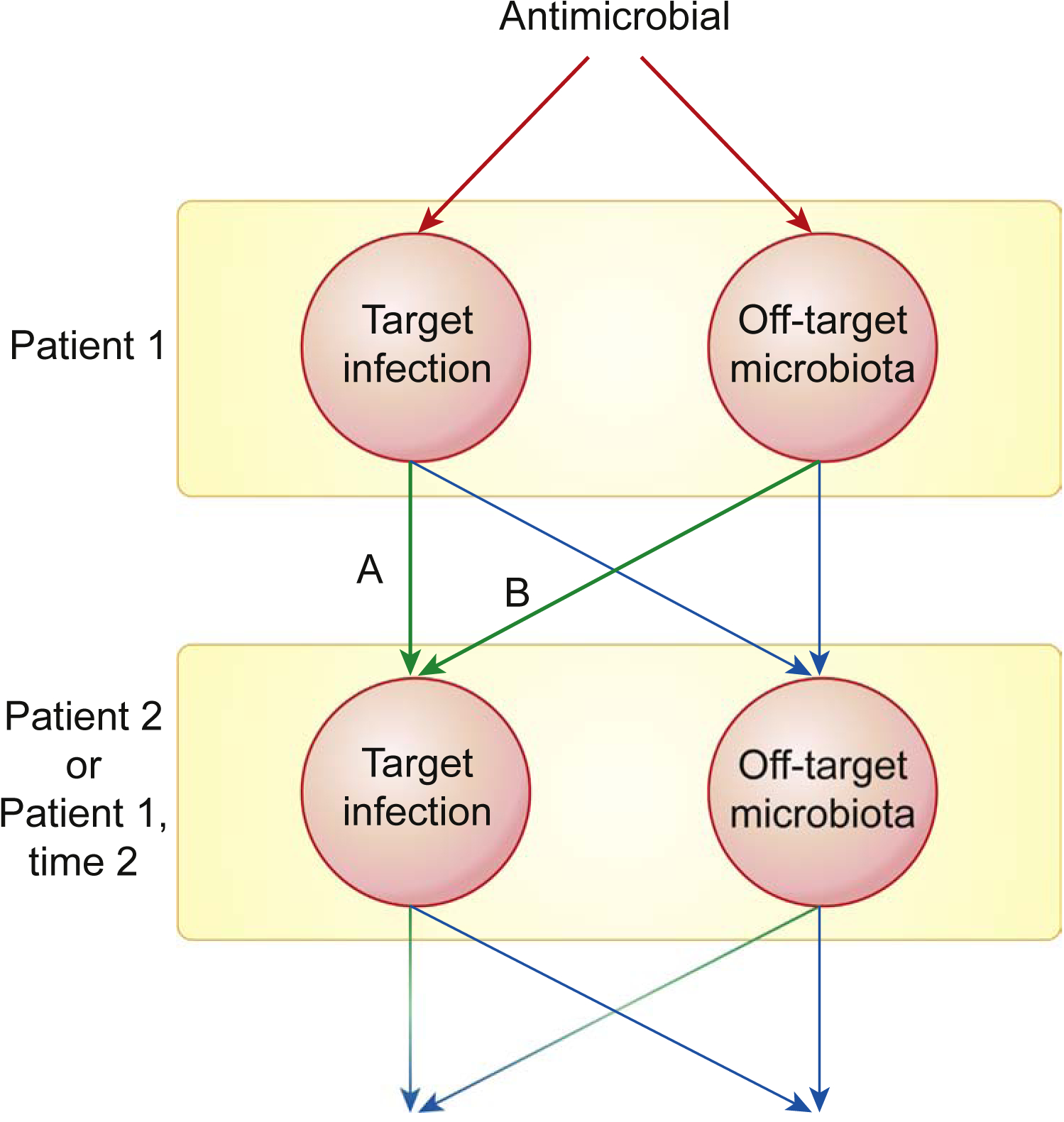
Antimicrobial Therapy Can Promote the Emergence of Drug Resistance in the Target Infection and in Off-Target Microbiota. Resistant microbes from either of these pools can cause future symptomatic infections in the initial patient or in another patient. Quantifying the relative importance of target versus off-target microbes as the source of resistant infections (arrow A versus arrow B) is critical to informing antimicrobial stewardship strategies. The relative importance of pathways A and B varies based on the ecology of the potential pathogen species.

Antimicrobial therapy can enrich for resistance in the target infection and in off-target microbiota. Resistant organisms from either of these groups can subsequently cause infections in the treated patient or in other people (Figure 3, pathways A and B). As a result, prior antimicrobial exposure may heighten the risk for subsequent resistant infections. We could find no data relating increased resistance in off-target populations to subsequent resistant infections in other patients. However, attempts have been made to relate the risk of antimicrobial-resistant infections in a patient to earlier use of antibiotics by that patient. In some cases, these data strongly implicate prior off-target exposure (Figure 3, pathway B) as a risk factor for resistant infections.

Perhaps best studied is the risk of resistant urinary-tract infections (UTIs). Here, the infecting organism often originates from the patient’s GI flora (Figure 3, pathway B) [72]. Multiple case–control studies have concluded that prior antimicrobial exposure increases the risk for resistant UTIs [reported odds ratio (OR) range 1.1–20.6], especially when the prior exposure occurred within 3 months of the infection [73–80]. This could be due to relapse with resistant target bacteria (Figure 3, pathway A) or from resistant bacteria selected in the GI tract (Figure 3, pathway B). Disentangling these possibilities requires knowing the target of the prior antimicrobial prescription. We know of only two studies that have carefully looked at this [77,79].

One case–control study considered 903 adult primary-care patients with UTIs caused by *E. coli* [77]. Patients with ampicillin-resistant UTIs were significantly more likely to have had a course of amoxicillin lasting ≥7 days in the previous month [OR 3.9, 95% confidence interval (CI) 1.64–9.34] or the previous 2–3 months (OR 2.29, 95% CI 1.12–4.70) compared with patients with ampicillin-susceptible infections. Previous amoxicillin treatment had most often targeted respiratory-tract infections, in which case effects on bacteria in the digestive or urinary tracts was off-target (Figure 3, pathway B). Another study considered 533 pediatric outpatients at their first UTI diagnosis, and evaluated the relationship between antimicrobial use in the 120 days prior to diagnosis and resistance [79]. That prior antibiotic use was aimed at a variety of non-UTI ailments, most commonly respiratory-tract infections. Amoxicillin use in the 30 days (OR 3.6, 95% CI 1.6–8.2) and 31–60 days prior to infection (OR 2.8, 95% CI 1.0–7.5) was associated with ampicillin-resistant UTIs. Exposure to amoxicillin N60 days before the UTI was not associated with resistance. These data suggest that antimicrobials targeting respiratory-tract infections enriched resistant off-target bacteria in GI tracts, and these bacteria subsequently initiated resistant UTIs (Figure 3, pathway B). In both of these UTI studies, this enrichment could be from antibiotic selection exerted on the pre-existing flora or from favoring colonization with resistant, rather than sensitive, exogenous organisms.

In hospitals, antimicrobial exposure increases risk for resistant nosocomial infections possibly originating from the patient’s own flora. Case–control studies consistently report that recent antimicrobial exposure is a risk factor for carbapenem resistance in hospital-acquired *Acinetobacter baumannii* [81,82], *K. pneumoniae* [83–87], and Enterobacteriaceae [88] infections. These studies considered antimicrobial use within 6 months prior to infection. Interestingly, carbapenem exposure increased the risk for carbapenem resistance (reported OR range 1.83–5.22), but so did fluoroquinolones (OR 1.87–4.54), cephalosporins (OR 2.55–2.87), and penicillins (OR 1.15–2.57) [82,84–88]. Prior antimicrobial exposure may have selected for conjugative plasmids or cellular mechanisms (i.e., upregulation of efflux pumps) conferring multidrug resistance [88]. These studies did not report the target of prior antimicrobial therapy; however, most included only the first occurrence of infection with the focal pathogen [81–85], implying that off-target exposure (Figure 3, pathway B) contributes to increased risk for carbapenem-resistant nosocomial infections. Similar studies found that prior antimicrobial use elevates risk for vancomycin resistance in enterococcal infections [89], methicillin resistance in nosocomial *S. aureus* infections [90], colistin resistance in bloodstream *K. pneumoniae* infections [91], carbapenem resistance in *P. aeruginosa* [92], resistance in invasive pneumococcal disease [93], and resistance in infections caused by Gram-negative bacteria [94].

Studies investigating the connection between antimicrobial usage and resistance are challenging to design and interpret [95–97]. When individuals with susceptible infections serve as controls, an association between antimicrobial use and resistance may indicate that antimicrobial use decreases the likelihood of infection with susceptible pathogens rather than increasing the likelihood of infection with resistant pathogens. Additionally, the heterogeneity of existing studies, including methods for measuring antimicrobial exposure, methods for measuring susceptibility of bacteria, and inclusion criteria for patients, makes these studies challenging to compare [97]. Thus, a direct causal link between antimicrobial exposure and resistant infections is difficult to establish. Randomized trials and prospective cohort studies could enable more rigorous understanding of the connection between exposure and resistance [96].

Despite these challenges, the preponderance of available evidence supports a correlation between antimicrobial exposure and increased risk of resistant infection in individual patients, and it seems likely that off-target exposure contributes to this risk. The evidence suggests that elevated risk runs from marginal to tenfold or more, and that the window of elevated risk is generally brief, lasting for a few weeks or months following exposure.

## Antimicrobial Stewardship

The importance of off-target exposure will vary for different pathogen species, which necessitates different stewardship strategies. For some pathogens, resistant organisms are most likely to transmit or recur from target populations (Figure 3, pathway A). For example, an estimated 85% of the cephalosporin exposure experienced by *Neisseria gonorrhoeae* occurs when *N. gonorrhoeae* is the target of treatment [2]. Therapy targeting gonorrhea likely drives the spread of cephalosporin resistance in *N. gonorrhoeae* (Figure 3, pathway A), although off-target exposure also plays a role [98,99]. For other pathogens, off-target antimicrobial exposure might be the primary driver of resistance evolution (Figure 3, pathway B). There is no one-size-fits-all stewardship strategy; the best strategy will depend on the relative importance of pathways A and B for a given organism and drug. When pathway A is more important for a patient’s future health, controlling resistance will depend on managing target exposure through dosing and drug choice. Managing resistance in COPs like *Klebsiella* (Table 1), where pathway B plays an important role, requires managing off-target exposure.

Even when pathway B is the most important, the poor understanding of the magnitude of the absolute risks involved makes implementing rational stewardship policies hard. Take the simple case of a respiratory-tract infection caused by a virus. Here, there are no concerns about pathway A evolution, and avoiding antibiotic therapy altogether is the best way to minimize off-target resistance evolution. But there is a risk associated with doing that (for instance when diagnosis is uncertain, or there is a possibility of secondary bacterial involvement). This means that the risk to patient health associated with withholding treatment needs to be balanced with the risk to future health caused by resistance evolution. All the evidence we have reviewed above shows that off-target antibiotic exposure can cause resistance problems in the future, but all that evidence concerns relative risk, not absolute risk. How to balance up to tenfold risks over relatively brief windows (at least in outpatient populations) against the absolute risks of not treating now? The problem gets even harder if we want to factor in risk to others from transmission. We know that risk is not zero, but little more than that.

**Figure 4.**
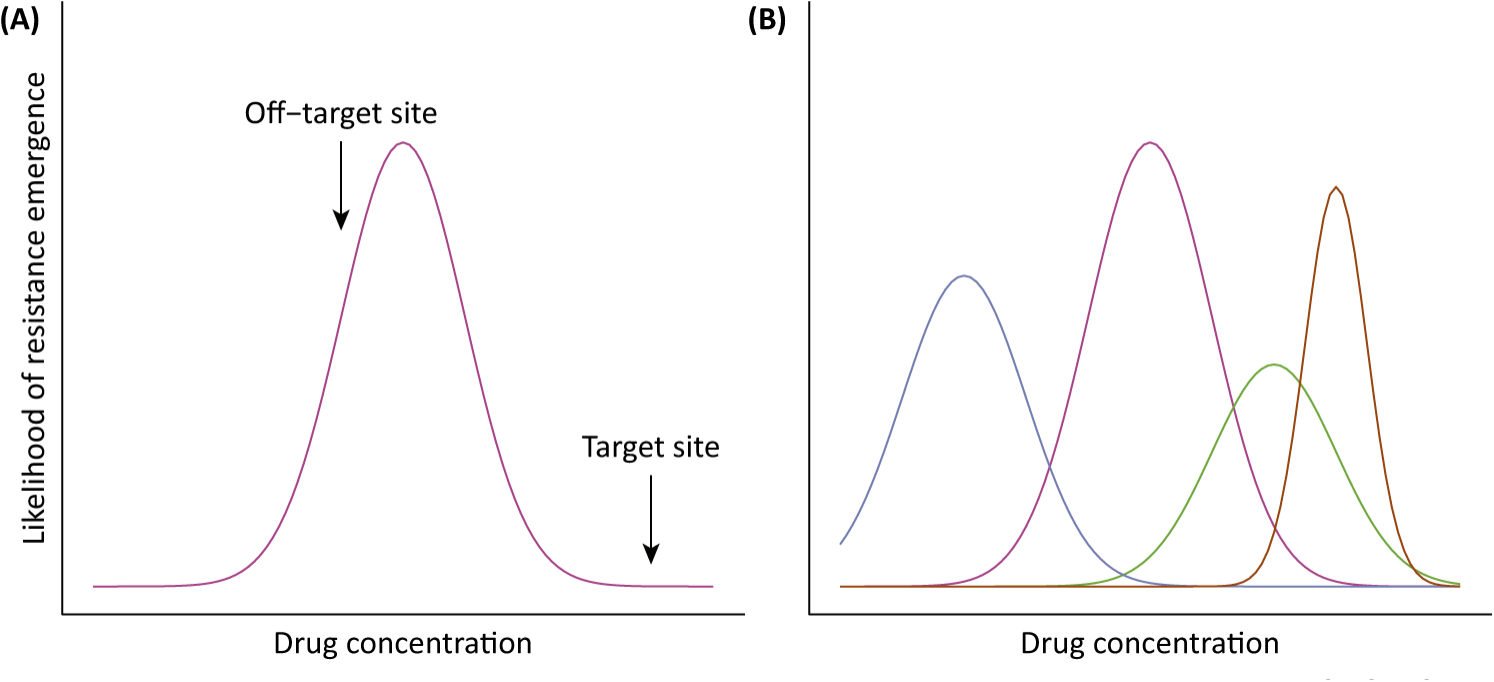
Stewardship Challenges in Antimicrobial Dosing. (A) Likelihood of resistance emergence in a given species is maximized at intermediate drug concentrations [101]. Note that these curves can have different shapes if, for example, more than one resistance mechanism can arise. During antimicrobial treatment, different drug concentrations occur at different anatomical sites. Therefore, optimizing dosing to reduce the likelihood of resistance emergence across different sites may not be possible. (B) When multiple species co-occur at an anatomical site (colors), the same drug concentration may have different impacts on resistance emergence in each species. Therefore, it may be challenging to choose a dose that minimizes the likelihood of resistance emergence in all species.

Another simple case that illustrates how much we cannot yet rationally determine is the question of optimal drug doses to limit resistance emergence. The dose administered determines the drug concentration bacteria experience, thereby influencing the likelihood of resistance emergence. With no drug, there will be no selection for resistance, and if the dose is high enough to kill every microbe, resistance will not also evolve. Between those extremes, resistance will evolve (Figure 4A) [100–102]. Efforts to optimize dosing typically focus on target organisms. However, targets and off-targets often exist at different anatomical sites where pharmacokinetic processes produce different drug concentrations. Therefore, it might be impossible to find a dose that would minimize resistance emergence optimally in both targets and off-targets (Figure 4A). When trade-offs exist between preventing resistance emergence in targets and off-targets, understanding the relative importance of these two groups (pathway A versus pathway B) can be critical to decision-making. At sites with diverse microbial communities, the same drug concentration might affect resistance emergence differently in different species or strains (Figure 4B), in which case decision making would require prioritizing species that pose the greatest absolute risks to future health, information which is currently largely lacking. In some cases, these trade-offs between targets and (most) nontargets can be circumvented, for instance, by exploiting administration routes that target antimicrobials locally rather than systemically (e.g., topical application for an infected wound).

Stewardship policy can also encourage the development and preferential use of antimicrobials with fewer off-target effects. For example, updated international guidelines for treating uncomplicated UTIs discourage the use of fluoroquinolones in favor of antibiotics such as nitrofurantoin, which minimize collateral damage to the microbiome [19,103,104]. Nitrofurantoin is rapidly excreted renally and only reaches high concentrations in the urine [105]. Narrow-spectrum drugs that target only a subset of microbial taxa can also be developed and preferentially used. Narrow-spectrum drugs can minimize off-target resistance evolution because they impose selective pressure only on a subset of the microbiota. For example, the bacteriocin thuricin CD kills *C. difficile* as effectively as vancomycin, but it has a lesser impact on bystanders in the intestinal microbiome due to its narrow host range [106]. However, culturing and determining antimicrobial susceptibility for an infectious agent takes time, and during this window broad-spectrum antimicrobials can be life-saving for patients with severe infections. Rapid diagnostic tools could facilitate more widespread use of narrow-spectrum drugs [107]. A comprehensive rapid diagnostic strategy should include characterizing carriage of COPs and antimicrobial resistance in off-target populations as well as in the focal infection [108]. A related strategy, de-escalation, recommends starting a patient on a broad-spectrum antimicrobial and then moving to a more narrow-spectrum drug once the infectious agent has been identified and described. In theory, starting with a broad-spectrum antimicrobial minimizes the initial risk of inadequate antimicrobial therapy, and then switching to a narrow-spectrum antimicrobial minimizes off-target effects. Currently, there is insufficient evidence to evaluate whether de-escalation practices have an impact on the frequency of antimicrobial resistance [109,110].

Further complicating the problem, many commonly used nonantibiotic medications select for antibiotic resistance. One study found that 24% of 1000 tested nonantibiotic medications inhibited growth of gut bacteria *in vitro* [111]. Troublingly, the evolution of resistance to these nonantibiotic drugs correlated with increased resistance to antibiotics. Incorporating management of nonantibiotic drugs into antimicrobial stewardship may be necessary.

## Novel Strategies

Ideally, target pathogens would be treated while minimizing off-target exposure. New adjunctive therapies that locally inactivate antimicrobials at off-target sites may make this possible. Development of these adjuvants has focused on preserving the intestinal microflora by site-specific antimicrobial inactivation without altering plasma drug concentrations. Early successes have been achieved with orally administered β-lactamases given with intravenous β-lactam antibiotics. β-lactamases enzymatically inactivate β-lactams. Under the name SYN-004, this β-lactamase treatment advanced to clinical trials in human subjects [112–115]. Data from clinical trials show that the drug successfully inactivates β-lactams in the digestive tract without adversely affecting levels of antibiotic in plasma [112–115]. In animal models, this protects against loss of intestinal species richness and against resistance-gene enrichment [116]. An alternative adjuvant is activated charcoal encased in zinc-pectinate beads. Activated charcoal sequesters antimicrobials through adsorption rather than relying on enzymatic inactivation, which means that this strategy could be effective with a broad range of antibiotic classes [117]. The zinc-pectinate beads are also compatible with orally administered antimicrobials when the target treatment site is outside the intestines. The beads encasing the charcoal remain intact in the small intestine, where orally administered antimicrobials need to be absorbed to reach the target site, and then the beads release activated charcoal in the colon, sweeping up any remaining antibiotic [117]. DAV132, a recent formulation of the beads, was shown to site-specifically bind antimicrobials in a Phase I clinical trial [118,119]. Activated charcoal beads and β-lactamases present two promising strategies for reducing selective pressure on off-target bacteria in the microbiome without compromising treatment of target bacteria.

## Concluding Remarks

Available data support the precautionary principle for limiting off-target antimicrobial exposure, but quantitative understanding of the risks posed to patient health are lacking. Different strategies are required to manage resistance in target and off-target populations, and it may be impossible to optimize outcomes in both groups. Designing optimal antimicrobial stewardship programs requires knowing the relative risks associated with target versus off-target antimicrobial exposure. The magnitude of these risks is likely to vary among patient populations, partly because some patients are more likely than others to suffer a serious infection in the weeks following antimicrobial therapy (i.e., seriously ill hospital patients versus otherwise healthy outpatients). Even less is known about how off-target exposure contributes to the spread of resistant organisms among patients, which is a key knowledge gap (see Outstanding Questions). Understanding these risks might require extensive genetic screening of infecting and colonizing organisms in patients before and after antimicrobial therapy to uncover patterns of migration and gene flow between the microbiome and infected sites, the relative contribution of expansion and acquisition of resistant genotypes in the microbiome during treatment, and the rate at which resistance in the microbiome decays following treatment. Defining the size of the problem, as well as the ecological and evolutionary processes that generate it, are critical to informed stewardship decision-making. There is also an urgent need for hard data directly connecting resistance evolution due to off-target exposure in one patient to onward transmission of resistant pathogens to others, and the associated health risks. Quantifying the contribution of off-target exposure to resistant infections is critical to designing rational stewardship policies, but there is a long way to go.

## Figures and Tables

**Table 1.** Colonizing Opportunistic Pathogens (COPs)

Pathogen	Colonization site	Are colonizing populations a source of future infections within a patient?	Refs
*Candida albicans*	GI tract, mucosal surfaces	Yes	[42]
*Enterobacter* spp.	GI tract	Yes	[49]
*Enterococcus* spp.	GI tract	Yes	[43]
Extraintestinal pathogenic *Escherichia coli* (ExPEC)	GI tract	Yes	[40]
*Haemophilus influenzae*	Respiratory tract	Yes	[44]
*Klebsiella pneumoniae*	GI tract, throat, nasal cavity	Yes	[35,36]
*Pseudomonas aeruginosa*	GI tract, skin	Yes, but environmental reservoirs are likely more important	[50,51]
*Staphylococcus aureus*	GI tract, nasal cavity, skin	Yes	[38,39,41]
Coagulase-negative staphylococci (CoNS)	Skin, mucosal surfaces	Yes	[52]
*Streptococcus pneumoniae*	Upper respiratory tract	Yes	[33]

## Highlights

Antimicrobial therapy enriches for resistance in off-target microbiota.

For some pathogen species, off-target antimicrobial exposure may be a major driver of antimicrobial resistance.

Antimicrobial therapy is a risk factor for subsequent infection with resistant organisms. Enrichment for resistance in off-target microbiota likely contributes to this risk.

Off-target antimicrobial exposure can be managed through traditional stewardship methods like reducing unnecessary antibiotic prescriptions, but quantitative data necessary to optimize patient treatment and evolutionary risk mitigation are generally lacking.

Novel adjunctive therapies that shield the microbiome from antimicrobials offer one way to mitigate off-target selection.

## Outstanding Questions

How often do antimicrobials reach concentrations that select for resistance at relevant off-target sites? How variable is this among patients?

What is the ecology of COPs in the community and healthcare settings? What are the patterns of migration and gene flow among anatomical sites and among patients? How often are colonizing resistant populations sources of resistant infections?

What are the barriers to HGT in the microbiome? How often are resistance genes transferred between commensals and pathogens? What controllable factors affect rates of transfer (drug concentrations, sizes and densities of donor, and recipient populations)?

How long does resistance persist in the microbiome after antimicrobial therapy? How do treatment duration, patient population, environmental factors, etc. influence persistence?

How does off-target exposure contribute to the spread of resistant organisms between patients?

What is the relative contribution of antimicrobial exposure on target versus off-target populations to clinically relevant antimicrobial-resistant infections?

Would strategies to limit the impact of antimicrobials on the microbiome reduce the incidence of clinically relevant antimicrobial-resistant infections at the population level?

## Glossary

Antimicrobial stewardship: strategic use of antimicrobials to optimize patient outcomes while minimizing the spread of antimicrobial resistance.
Bystanders: microbes other than the targets of treatment.
Colonization resistance: the phenomenon in which intact microbiota prevent colonization by other microorganisms.
Colonizing opportunistic pathogen (COP): an organism that colonizes its host asymptomatically, but can cause infections when introduced to other anatomical sites or immunocompromised hosts.
Commensal: an organism that benefits from its association with a host, while the host incurs no benefit nor cost.
Conjugation: the horizontal transfer of genetic material directly through cell-to-cell contact.
Horizontal gene transfer (HGT): the transfer of genetic material between organisms by a mechanism other than descent.
Microbiota/microbiome: the microorganisms in a particular environment, such as the human body.
Off-target: not the target of antimicrobial therapy.
SOS response: a bacterial response to DNA damage that alters expression in a network of genes.
Target: the population that antimicrobial therapy aims to eliminate or suppress.
Transduction: the horizontal transfer of genetic material facilitated by bacteriophages.
Transformation: horizontal genetic transfer via uptake of exogenous genetic material.
